# Gender reporting across regions and time in psychological studies: a scoping review of studies in *Psychological Science* between 2019 and 2024

**DOI:** 10.1186/s41073-025-00186-8

**Published:** 2026-01-20

**Authors:** Tiantian Chen

**Affiliations:** University of Oxford, University of Oxford, 1 St Giles, Oxford, OX1 3JS UK

**Keywords:** Gender reporting, Non-binary identity, Gender identity, Gender diversity, Psychology

## Abstract

**Background:**

Despite growing calls for gender-responsive psychological research, implementation of gender-related guidelines is underresearched. The Sex and Gender Equity in Research (SAGER) guidelines recommend reporting participants’ gender, presenting gender-stratified results, analyzing gender-related data, acknowledging non-binary identities, and distinguishing between biological sex and social gender. This scoping review assessed the extent to which these guidelines are followed.

**Methods:**

We included all primary data studies on human participants published in *Psychological Science* from 2019 to 2024 (*n* = 699) and assessed their gender reporting practices according to the SAGER guidelines.

**Results:**

While 87.8% (*n* = 614) of studies reported participants’ gender, only 35.3% (*n* = 247) presented gender-stratified results, and 24.2% (*n* = 169) conducted gender-based analysis. Only 17.2% (*n* = 120) of studies reported participants’ non-binary identities. Regional patterns emerged: Global North studies more frequently reported non-binary identities but less often presented gender-stratified results and conducted gender-based analysis than Global South studies. The U.S.-based studies saw a notable decline in reporting gender-stratified results, from 43.2% (*n* = 32) in 2022 to 28.1% (*n* = 16) in 2024.

**Conclusion:**

This review reveals persistent inconsistencies in how gender is conceptualized and reported. It provides recommendations to improve gender reporting in order to facilitate the production of more accurate and socially relevant knowledge in psychological research.

**Supplementary Information:**

The online version contains supplementary material available at 10.1186/s41073-025-00186-8.

## Background

Gender influences psychological experiences across the lifespan. Neglecting it in research risks producing inaccurate findings and incomplete theories. Psychology has historically overlooked gender and particularly underrepresented women in research design. For example, early studies often relied on convenience samples composed primarily of White, college-educated men [32, 58]. Indeed, Holmes and Jorgensen [45] found that male participants were twice more than female participants in three psychological journals in 1966. Common reasons for single-gender recruitment included limited awareness of gender differences in hypotheses, easier access to male participants, cultural hesitancy about recruiting female participants, and funding priorities [66]. Generalizing findings from male-only samples to all humans could obscure potential gender difference and compromise the accurate understanding of human behavior. Nowadays, while awareness of gendered sampling bias has grown [20], persistent disparities remain. For example, Walton et al. [74] found that sport psychology continues to over-represent male participants. And Lefler et al. [50] found many researchers who value gender as an important demographic variable do not incorporate it into their own work. Even when gender data are collected, studies often fail to report or analyze them [24]. These findings suggest that male-centered perspectives and inadequate gender analysis remain embedded in contemporary psychological research.

Moreover, beyond binary categories, gender is increasingly conceptualized as a spectrum [41]. Diverse genders have existed across different historical periods and cultures [40, 57], yet many psychological studies either ignore or mis-classify them. For example, evolutionary psychology often frames gender as biologically determined [62]. This view reinforces the idea that there are only two biologically and behaviorally distinct categories: male and female. Psychological surveys typically offer only “male” and “female” options [26], and such binary framing can misrepresent gender diverse individuals [65]. This exclusion not only invalidates these people’s experiences but also leads to inaccurate data collection.

In response, in the past decade, several guidelines encourage researchers to incorporate gender in psychological research by treating it as a key demographic variable and analyzing gender-related data when appropriate. One major step in this process is transparent reporting of gender data and precise use of gender-related terminology. To promote transparent gender reporting, for example, the American Psychological Association (APA) [2] encourages researchers to report participants’ gender identities. Similarly, the Sex and Gender Equity in Research (SAGER) guidelines [37] emphasize collecting gender identity data during sample recruitment and reporting gender information in research samples, results, and data analysis [30]. To promote conceptual clarity in gender reporting, both the APA [1] and the SAGER guidelines encourage researchers to distinguish between gender as a socially constructed concept and biological sex. Additionally, the APA [3, 4] warned against assuming participants’ cisgender identities. Though the SAGER guidelines do not explicitly address non-binary identities, they urge researchers not to reduce gender to a male/female binary [37]. Cameron and Stinson [16, 17] and Lowik et al. [52, 54] further developed practical recommendations for inclusive and diverse gender classification terms. Broussard et al.'s [13] findings support these approaches, as they found both cisgender and gender-diverse participants preferred non-binary gender question formats. Together, these efforts promote more ethical, rigorous, and inclusive research practices.

Most of these research guidelines were published by institutions in the U.S. or Europe. As Bhatia [10] contends, the globalization of psychology often involves disseminating American psychological theories and epistemological perspectives as universal truths rather than foster a more equitable and inclusive approach that acknowledges American psychology as one among many culturally specific knowledge systems. Some countries, particularly in the Global South, may lack similar institutional mandates for gender related research practices. While some studies highlight these regional disparities [12], we know little about how institutional policies, cultural attitudes toward gender, and adherence to research guidelines vary across countries and influence gender reporting in psychology.

This scoping review provides a descriptive analysis of whether and how researchers report gender in sampling, results, and analysis in primary data studies published in *Psychological Science* between 2019 and 2024. It also explores whether these research practices vary across countries. It uses the SAGER guidelines as a benchmark. Rather than test hypotheses, this review aims to describe current practices and identify areas for improvement to enhance transparency and inclusivity in psychological research. Two concerns often surface in debates on gender reporting. First, some argue that gender reporting is unnecessary unless hypothesized as relevant. Second, some question whether gender-based analysis is necessary when gender is not central to the research question. However, even when gender is not a primary variable, transparent reporting of participants’ gender allows other researchers to assess the generalizability of findings. This review therefore assesses whether gender data were reported and discussed once they were collected. This review does not evaluate the validity of individual analysis. Instead, it examines whether gender is reported in ways that support transparency and precise use of gender related terms.

Previous reviews, conducted before 2020, have addressed specific aspects of gender reporting, such as whether gender was mentioned in the title, abstract, or participant section [35], whether it was included in analysis [59], and whether diverse gender identities were reported in research samples [17]. Cavanaugh and Abu Hussein [18] evaluated journal-level guidelines. However, they did not examine how the absence of such guidelines influenced actual research practices, nor did they conduct a country-level analysis. Given the time frame of these reviews, an updated analysis is needed to assess whether researchers have improved their practices in response to recent calls for more gender-responsive and inclusive research. By evaluating gender reporting in psychological research, this review aims to offer recommendations for more transparent and ethical research.

## Methods

To assess gender reporting practices in psychological research, we manually retrieved all empirical articles published in *Psychological Science* between 1 January, 2019 and 31 December, 2024 from the journal’s official website (https://journals.sagepub.com/home/pss). We chose *Psychological Science* because it is a leading journal of the Association for Psychological Science (APS). Although it does not mandate gender reporting, it encourages authors to use inclusive and bias-free language on gender according to APA guidelines [7]. We reviewed articles published from 2019 onwards to capture recent developments in gender reporting, especially following the publication of the SAGER guidelines and the APA guidelines. This time frame also complements earlier reviews, such as Cameron and Stinson [17], which examined similar issues in *Psychological Science* for earlier publication years.

The review was registered in Open Science Framework (OSF) at https://osf.io/86zcv.

Between 1 January, 2019, and 31 December, 2024, Psychological Science published 861 articles. We imported the bibliographic data into Excel. To minimize mis-classification, two reviewers screened titles and abstracts for each time period (TC and VO screened the same studies published between 1 January, 2019 and 31 December 2022; TC and YG screened the same studies published between 1 January, 2023 and 31 December 2024) to identify if studies met the inclusion criteria. Any discrepancies were resolved through discussion. And we read full-texts when needed.

**Fig. 1:**
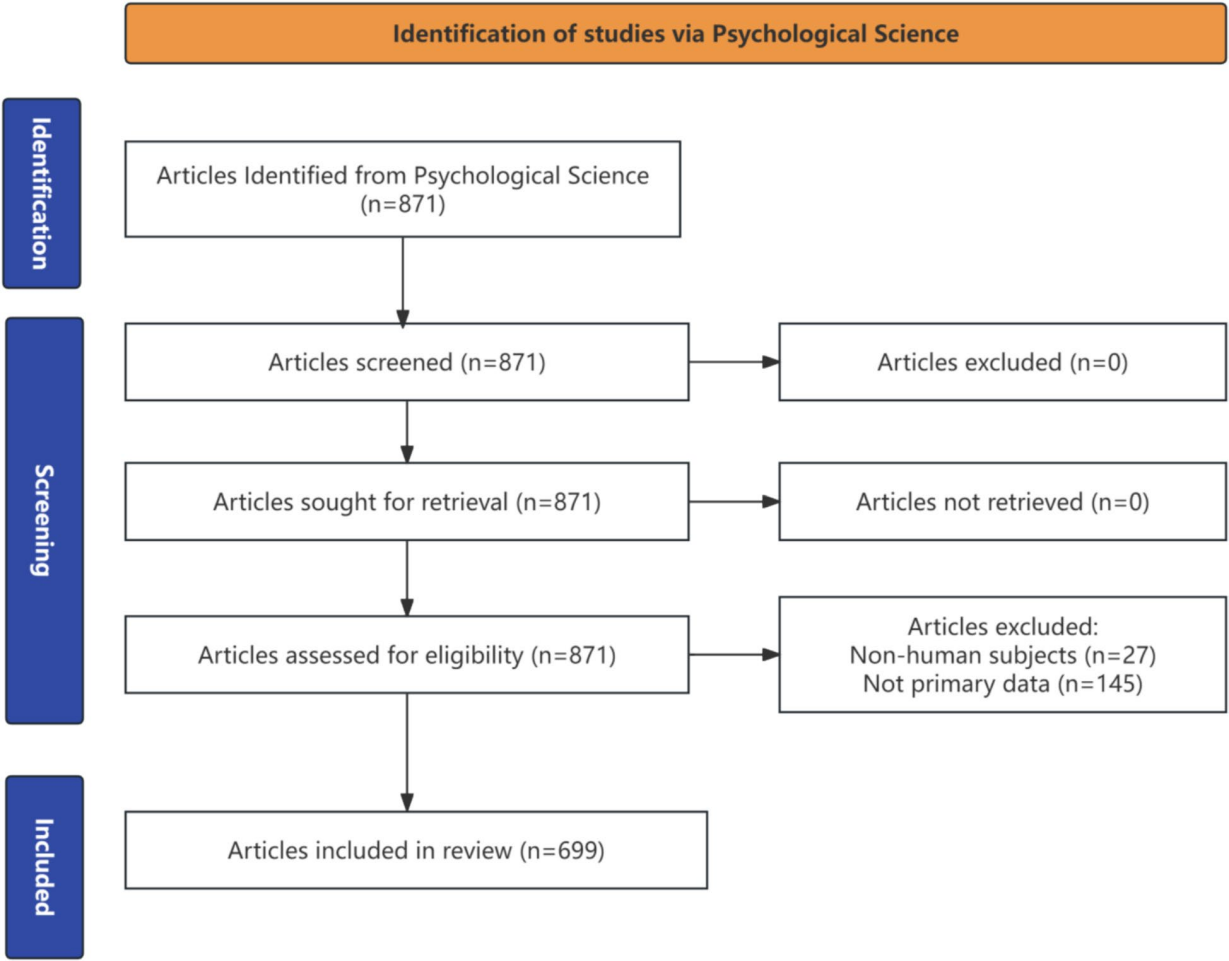
Preferred Reporting Items for Systematic reviews and Meta-Analyses (PRISMA) Flow Chart

Only articles reporting primary data about human participants were included in the review. We excluded meta analysis/systematic reviews (eg. [53]), commentaries (eg. [29]), and editorials (eg. [9]), as well as studies involving non-human participants such as chimpanzees (eg. [15]). Studies focusing on fetal development, such as Ustun et al. [71], which examined how fetuses respond to different flavors, were excluded, because reporting fetal gender is ethically sensitive or legally restricted in many countries [60]. Studies analyzing social media posts (eg. [38]) were excluded, but studies on social media users were included because gender is readily discernible in social network analysis. Figure 1 shows the Preferred Reporting Items for Systematic reviews and Meta-Analyses (PRISMA) flow chart for the screening process. We provide the PRISMA Checklist and the screening records in Additional File.

Two reviewers then assessed the methodological quality of the included literature for each time period using the Mixed Methods Appraisal Tool [44]: TC and VO assessed the same studies published between 1 January, 2019 and 31 December 2022, and TC and YG assessed the same studies published between 1 January, 2023 and 31 December 2024. The Mixed Methods Appraisal Tool uses standardized questions to assess the methodological quality of qualitative, quantitative, and mixed-methods studies. Subsequently, the reviewers created a pilot data extraction sheet on Excel based on the first five articles published in Psychological Science in January 2021. Extracted information included bibliographic data, study’s geographical location (by country), and any information related to gender, sex, or gender identity found in the articles. Given that this review focused on publishing ethics, only public data were collected. We did not contact authors for unpublished information. This approach allows us to evaluate the extent to which gender reporting is reflected in visible research outputs of the field. We identified the study’s geographical location as the country (or countries) where the institutions or ethics committees granting approval were based. This approach reflects the regulatory, funding, and cultural environments in which studies were designed. And it is particularly relevant for online studies, where participant recruitment may span multiple regions but ethical oversight was centralized within specific jurisdiction. For studies approved by multiple ethics committees, we recorded the location of each committee. These data were then used to group studies by region (Global North and Global South) to examine geographical patterns in gender reporting practices. Two reviewers collected data for each time period (TC and VO extracted data from the same studies published between 1 January, 2019 and 31 December 2024; TC and YG extracted data from the same studies published between 1 January, 2023 and 31 December 2024) and resolved discrepancies through discussion.

We evaluated gender reporting practices according to the SAGER guidelines. The SAGER guidelines recommend reporting participants’ gender, presenting results stratified by gender, and conducting gender-based analysis. The guidelines also advise distinguishing between biological sex and social gender and avoiding binary assumptions. According to these principles, we developed six evaluation questions (see Table 1).

**Table 1:** Evaluation Sheet

	**SAGER Recommendations**	**Evaluation questions**	**Examples of what counts**	**Notes**
1	Reporting participants’ gender information	Does the study report participants’ gender or explain why gender was not reported?	• “Reported the percentage of male/female participants in surveys” [21] • “Gender was not recorded due to a technical issue” [55]	For studies on couples, gender was counted as reported only when couples’ genders were specified. For single-gender samples, it was counted as reported when authors stated the gender of participants and justified the single-gender design
2	Presenting results stratified by gender	Does the study present results stratified by gender (e.g., reporting group means, proportions, or regression outputs that include gender), consider gender when reporting results, or briefly note the absence of gender-stratified data in the results section?	• “Provided Hierarchical Linear Model analysis results for men” [21] • “Gender as covariate in additional regression models to test the robustness of findings” [73]	Single-gender studies were coded as presenting results stratified by gender when the single-gender results were explicitly stated
3	Conducting gender-based analysis	Does the study interpret gender-related findings, explain their theoretical or empirical implications, or mention there is no need for such analysis?	• “Women may may experience greater postdisaster psychological stress according to experiment results” (Deng etl al, 2024) • “Gender was not included in the discussion due to statistically non-significant results.”[48]	For single-gender samples, studies were not coded as conducting gender-based analysis unless they included discussion comparing findings with other genders
4	Avoiding conceptualizing gender as a binary factor	Does the study report participants’ non-binary identities or provide reasoning for adopting a binary approach in the research design?	Binary: “men or women” Non-binary: “prefer not to say,” “other genders,” and etc	We focus on reporting rather than actual recruitment of non-binary respondents. This approach addresses a potential limitation of this review: we cannot determine which gender options were originally provided to participants. For example, if a study only reported the percentage of male and female respondents, this does not necessarily mean no other options were available—it might indicate that all respondents selected either men or women. However, when other options exist, we argue studies should explicitly report a count of zero for those categories, even if no respondents selected these options
5	Avoiding conceptualizing gender as a binary factor	Which terms does the article use to describe participants’ non-binary gender?	“Undisclosed,” “other gender,” “transgender,” and etc	
6	Distinguishing between biological sex and social gender	Which term does the article adopt?	“Gender,” “sex,” or “both gender and sex are mentioned”	

The first four evaluation questions assessed whether studies provided transparent information regarding gender, sex, or gender identity in the methodology, results, and discussion sections. They capture different levels of engagement with gender. Presenting gender-stratified data and conducting gender-based analysis are distinct aspects of gender reporting. The former refers to reporting practices that present or justify not presenting gender-related data in the results section without requiring interpretation of gender effects, while the latter reflects analytical engagement with gender, such as explaining observed differences, interpreting null results, or justifying the absence of such analysis. We adopted a broad definition of presenting gender-stratified results. We counted any consideration of gender in the results section, such as including gender as a covariate [73]. This aligns with the SAGER guidelines which encourage authors to consider the relevance of gender to their study findings whenever applicable. A study may fall into more than one category if it both presents gender-stratified results and conducts gender-based analysis. For example, we coded Hernandez et al. [39] as both presenting gender-stratified results and conducting gender-based analysis. In the results section, the authors reported excluding gender from their models due to multicollinearity, providing a transparent justification for this decision. Moreover, although gender was ultimately excluded for statistical reasons, this reflects active consideration of gender as a potential analytical variable and demonstrates methodological transparency.

This review used descriptive statistics to summarize gender reporting practices. We coded each study’s reporting status as a binary indicator variable. If the information was present, regardless of whether authors referred to it as gender, sex, or gender identity, we coded it as 1. If we could not find the relevant information or explanation in the published article or its supplemental materials, we coded it as 0. We did not assess whether gender was preregistered or added post hoc, since the focus of this review was the transparency and accuracy of gender reporting in published articles. The mean of this indicator represents the proportion of studies that reported gender information according to the SAGER guidelines. We calculated confidence intervals using the Wald method for a 95% confidence level. We derived confidence intervals to illustrate the plausible range of differences in reporting practices between regions (Global North and Global South) and over time rather than to assess statistical significance.

The final two questions assessed whether gender-related terminology was used appropriately and in line with SAGER recommendations when describing sex, gender, and non-binary identities. The fifth question examined how researchers described non-binary identities. These terms might include “gender non-binary,” “gender nonconforming,” “gender-creative,” “gender fluid,” and “genderqueer,” among others. The sixth question evaluated whether studies distinguished between social gender and biological sex. The SAGER guidelines advise authors to use “gender” when they refer to individuals’ identities and “sex” when biological distinctions are paramount [37].

## Results

A total of 699 articles met the inclusion criteria. Among these, 87.8% (*n* = 614) reported gender in sample description, 35.3% (*n* = 247) presented results stratified by gender, and 24.2% (*n* = 169) conducted gender-based analyses. A minority, only 17.2% (*n* = 120), took a non-binary approach to participants’ gender identity.

**Fig. 2:**
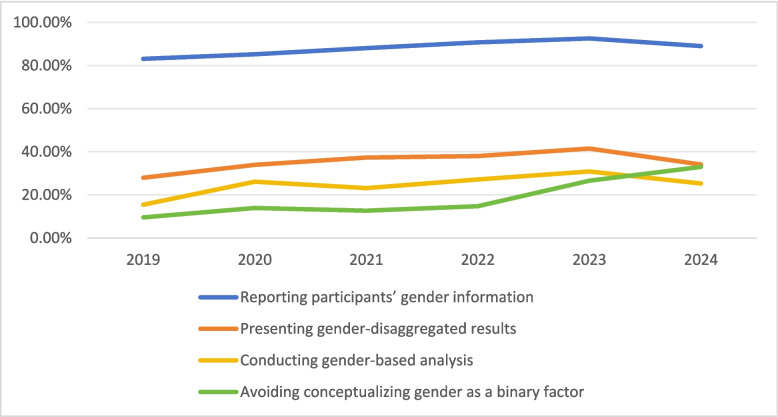
Gender reporting across time

The observed proportion of studies reporting participants’ gender increased from 83.1% in 2019 to 92.6% in 2023, before declining to 89% in 2024 (see Fig. 2 and Table 2). Reporting of results stratified by gender followed a similar pattern, rising from 27.9% in 2019 to 41.5% in 2023 and then decreasing to 34.1% in 2024. Gender-based analysis increased from 15.4% in 2019 to 30.9% in 2023 and then declined to 25.3% in 2024. These fluctuations suggest inconsistencies in gender reporting in psychological studies and underscore the need for ongoing monitoring. In contrast, reporting of non-binary identities increased clearly over time from 9.6% (*n* = 13) in 2019 to 33% (*n* = 30) in 2024.

**Table 2:** Gender reporting across time

	**2019**	**2020**	**2021**	**2022**	**2023**	**2024**
Number of studies included	136	115	134	129	94	91
Reporting participants’ gender information	113(83.1%)	98 (85.2%)	118(88.1%)	117(90.7%)	87 (92.6%)	81 (89%)
95% confidence interval	76.8%−89.4%	78.7%−91.7%	82.6%−93.6%	85.7%−95.7%	87.2%−97.9%	82.6%−95.5%
Presenting results stratified by gender	38 (27.9%)	39(33.9%)	50(37.3%)	50(38.8%)	39(41.5%)	31 (34.1%)
95% confidence interval	20.4%−35.5%	25.2%−42.6%	29.1%−45.5%	30.3%−47.2%	31.5%−51.5%	21.2%−40.3%
Conducting gender-based analysis	21 (15.4%)	30(26.1%)	31 (23.1%)	35 (27.1%)	29(30.9%)	23 (25.3%)
95% confidence interval	9.4%−21.5%	18%−34.2%	16%−30.3%	19.4%−34.8%	21.5%−40.2%	16.3%−34.3%
Avoiding conceptualizing gender as a binary factor	13 (9.6%)	16 (13.9%)	17 (12.7%)	19 (14.7%)	25(26.6%)	30 (33%)
95% confidence interval	4.6%−14.5%	7.6%−20.3%	7%−18.3%	8.6%−20.9%	17.6%−35.6%	23.3%−42.7%

**Fig. 3:**
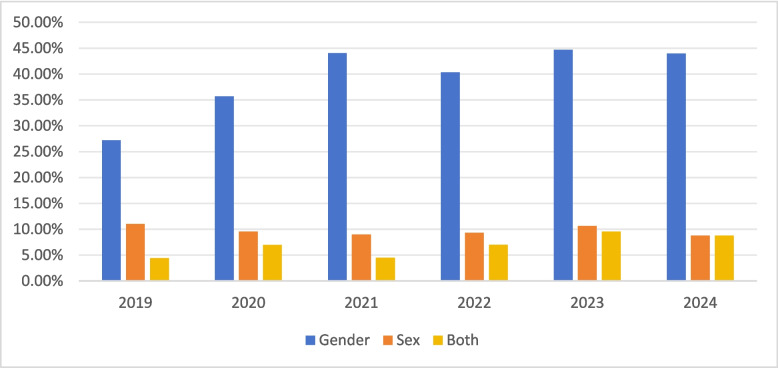
The use of gender terms across time

Across all years, researchers showed a strong preference for the term “gender” over “sex.” (see Fig. 3). Researchers employed various terms to depict gender diverse populations. The most frequently used term (appearing in 41 articles) is “undisclosed.” This term describes respondents who either declined or did not disclose their gender. For example, Schaumberg and Skowronek [69] included respondents who did not report gender in samples. The term “other” appears in 22 articles. For example, Moran et al. [61] found that less than 1% of respondents identified themselves with other identities. 17 articles used the term “non-binary.” For example, Feiler and Trede [28] included “non-binary” as an option alongside “male” and “female.” 15 articles utilized the term “transgender.” For example, Raines et al. [68] differentiated between transgender and cisgender individuals. Other terms included gender-nonconforming, intersex, the third gender, a different gender, and etc.

We categorized study location as Global North and Global South according to Finance Center for South-South Cooperation [27]. Two studies involved collaborations between countries from both regions and were therefore counted once in each category. As a result, the total regional count (*n* = 701) is slightly higher than the total number of studies included in the review (*n* = 699). 96.7% (*n* = 676) of studies originated from the Global North, while only 3.6% (*n* = 25) were from the Global South. Despite their dominance in publication volume, Global North studies lagged in gender reporting in the methodology, results, and discussion sections: 87.4% (*n* = 591) reported gender as a sample characteristic, 34.9% (n = 236) presented gender-stratified results, and 24% (*n* = 162) conducted gender-based analyses. In contrast, Global South studies reported higher rates at 100% (*n* = 25), 48% (*n* = 12), and 28% (*n* = 7), respectively. However, the Global North led in reporting non-binary identities. 17.6% (*n* = 119) of studies reported participants’ non-binary identities, compared with just 2.5% (*n* = 1) in the Global South.

Of 699 studies analyzed, the majority (55.1%) were from the U.S. Notably, between 2022 and 2024, the proportion of these studies that presented results stratified by gender declined from 43.2% to 28.1% (see Table 3). To better understand whether this pattern reflects a broader disciplinary trend or a region-specific dynamic, we compared U.S.-based studies with those from other Global North countries. The purpose of distinguishing U.S.-based from non-U.S. Global North studies was descriptive rather than inferential. This comparison provides contextual insight into reporting patterns within a journal where U.S. submissions constitute the majority. In 2024, 28.1% of U.S.-based studies and 48.1% of non-U.S.-based studies within the Global North reported results stratified by gender, compared with 43.2% and 29.4%, respectively, in 2022 (see Table 4). This suggests that U.S.-based research was less likely to present gender-stratified results in 2024. In previous years, this gap was smaller, with U.S.-based studies even surpassing their non-U.S. Global North counterparts in 2021 and 2022. Overall, this descriptive comparison highlights variation within the Global North and suggests that gender reporting practices may differ across research contexts.

**Table 3:** Gender reporting in the U.S.-based studies across time

	**2019**	**2020**	**2021**	**2022**	**2023**	**2024**
Number of studies involving the U.S. institutions	79	57	76	74	42	57
Reporting participants’ gender information	64 (81%)	48 (84.2%)	66(86.8%)	67(90.5%)	37(88.1%)	50(87.7%)
95% confidence interval	72.3%−89.7%	74.7%−93.8%	79.2%−94.5%	83.8%−97.3%	78.2%−98%	79.1%−96.3%
Presenting results stratified by gender	20 (25.3%)	18 (31.6%)	31(40.8%)	32(43.2%)	17(40.5%)	16 (28.1%)
95% confidence interval	15.7%−35%	19.4%−43.8%	29.7%−51.9%	31.9%−54.6%	25.5%−55.5%	16.3%−39.8%
Conducting gender-based analysis	13 (16.5%)	14 (24.6%)	19 (25%)	22(29.7%)	12(28.6%)	13 (22.8%)
95% confidence interval	8.2%−24.7%	13.3%−35.8%	15.2%−34.8%	19.2%−40.2%	14.7%−42.4%	11.8%−33.8%

**Table 4:** Comparison between U.S.-based studies and Global North studies that did not involve U.S. institutions on presenting results stratified by gender

	**2019**	**2020**	**2021**	**2022**	**2023**	**2024**
Global North studies that did not involve U.S. institutions	54	56	56	51	47	27
Presenting results stratified by gender	17 (31.5)%	19 (33.9)%	18 (32.1)%	15 (29.4)%	19 (40.4%)	13 (48.1%)
95% Confidence Interval	19%−44%	21.4%−46.4%	19.8%−44.5%	16.8%−42%	26.2%−54.6%	28.9%−67.4%

## Discussion

This review examined gender reporting in studies published in *Psychological Science* from 2019 to 2024. First, we found gender remains underreported and inconsistently analyzed in psychological research. While most studies reported participants’ gender, only a small fraction provide gender-stratified results, and even fewer conduct gender-based analysis. When gender is collected as a demographic characteristic but omitted from the results, it limits readers’ ability to understand whether findings apply equally across groups and risks obscuring potential gender-related effects. Even when gender-based analysis is not central to a study’s aim, explicitly stating that gender was considered or explaining why it was deemed irrelevant enhances research transparency and accountability. Conversely, omitting such information can lead to tokenistic reporting, where gender data are collected but not meaningfully integrated into the study’s interpretation. Admittedly, many studies are powered for the total sample rather than for gender-specific analysis. This means subgroup comparisons are often underpowered and may yield non-significant results for statistical reasons. This review does not call for unhypothesized or underpowered gender-specific analysis, nor does it advocate searching for gender differences without justification. Rather, it emphasizes that decisions about reporting gender-stratified results or discussing gender implications should not depend solely on statistical significance. Clearly reporting the study base and any gender-related considerations allows readers to better assess the generalizability and validity of psychological findings.

Inadequate gender reporting can also have broader conceptual consequences, as it may obscure or distort findings. When gender differences are a central focus, studies should be adequately powered to detect them; when they are not, transparent reporting of gender distributions remains essential for accurate interpretation. Failure to do so can lead to what Hare-Mustin and Marecek [36] describe as “beta bias” (ignoring differences) or “alpha bias” (overstating them). Research on children’s behaviors and abilities, for instance, has identified both gender differences and similarities [49]. When such findings are not transparently reported, readers may misinterpret them. They may attribute to unreported differences to researchers’ neglect and unreported similarities to stereotypical assumptions about gender [46]. To avoid the misunderstandings, researchers are encouraged to report gender-related findings transparently, explain any decision not to conduct such analysis, and reflect on how gender might have influenced the results.

Second, the percentage of reporting non-binary identities rose from 9.6% in 2019 to 33% in 2024.The increase in reporting non-binary identities from 2019 to 2024 suggests gradual progress toward more inclusive conceptualizations of gender. Continued monitoring beyond 2024 will be important to determine whether this trend persists, particularly as policy environments affecting gender-related data collection continue to evolve. Yet, most studies still adhere to a binary framework. Some researchers may exclude non-binary categories due to a lack of respondents identifying as such, but this omission is problematic. Other barriers include uncertainty around appropriate terminology[8], fear of government reprisal [43], and privacy concerns [63]. A binary approach prevents researchers from determining whether non-binary individuals participated, potentially erasing their presence from the study. For non-binary respondents, being forced to choose between “male” or “female” can feel awkward [47]. Consequently, they might skip the question [70] or select an inaccurate option under pressure [8]. Ultimately, assigning participants to the wrong experimental condition will compromise study reliability [42].

This review found that “undisclosed” was the most commonly used term for non-binary identities, followed by “other,” “non-binary,” and “transgender.” However, these terms can be inadequate. For example, “undisclosed” may imply that non-binary individuals either cannot or do not wish to identify themselves openly [6]. “Other” reinforces non-binary individuals’ marginalization and frames their identities as deviations from a presumed norm [8]. Transsexual individuals may reject being classified as transgender [31]. Emerging identities such as pangender, genderqueer, and agender further complicate classification. And gender fluidity makes capturing transitioning identities difficult [23]. Best practices recommend including non-binary and gender-diverse options rather than relegate them to residual categories [37]. Open-ended gender questions that allow participants to self identify with their own terminology can also foster inclusivity and reduce the risks of predefined categories [17]. Additionally, distinguishing between respondents who actively disclose a gender identity and those who select “prefer not to say” can promote more accurate and respectful representation of gender diversity in research [17].

Third, this review observed notable regional disparities in gender reporting. Institutions in the Global North have established gender reporting guidelines (e.g., the SAGER guidelines and the APA guidelines). However, our review found that Global North studies reported non-binary identities more frequently but lagged in reporting gender in the methodology, results, and discussion sections, whereas Global South studies were more likely to report gender in the methodology, results, and discussion sections but rarely reported non-binary identities. In addition, between 2022 and 2024, the proportion of the U.S.-based studies that presented results stratified by gender declined from 43.2% to 28.1%, whereas the proportion among non-U.S. Global North studies increased from 29.4% to 48.1%. This finding suggests that the decline of reporting results stratified by gender in 2024 may be disproportionately driven by the U.S.-based research. One possible factor contributing to the decline in the U.S. is the broader sociopolitical shift away from equity, diversity, and inclusion (EDI) policies. In 2024, over 30 states in the U.S. introduced or passed laws restricting EDI initiatives [5]. Though the policy change was enacted after 2024, this review raises concerns about earlier cultural and institutional shifts in U.S. research environments that may have preceded or anticipated the formal policy change. To understand the influence of policy change on gender reporting, continued monitoring of gender reporting practices in the U.S. context will be essential to assess whether these trends persist.

While overall Global South countries excel in gender reporting, important disparities remain. On the one hand, only 3.6% (*n* = 25) studies in our review originated from the Global South.

This under-representation [12, 33, 64, 67], likely reflects both lower research output and reduced publication opportunities [34, 51] compared with the Global North. Such imbalance may limit the external validity of our findings and underscores the need for more regionally diverse research on gender reporting in psychology.

On the other hand, only one study from the Global South reported non-binary identities. This under-reporting may stem from legal,cultural, and institutional barriers. Many Global South countries officially recognize binary gender categories [25]. Patriarchal and heteronormative structures in many Global South societies contribute to discrimination against gender-diverse individuals [72]. Difficulties in defining non-binary identity across diverse cultural contexts reinforce gender binarism [63]. For example, Indian law recognizes non-binary individuals as neither male nor female, but it does not provide an inclusive terminology for gender diversity [11]. Colonial legacies have also constrained traditional gender diversity. For example, hijras in Hindu culture were historically accepted as a third gender but were marginalized under British rule [22]. Moreover, while Global North frameworks like the SAGER guidelines conceptualize gender as a spectrum, these approaches may not align with local understandings and are sometimes perceived as imposing Western norms [19, 56]. Addressing these disparities requires developing context-specific best practices for gender data collection, expanding legal recognition of gender diversity, and strengthening institutional support for Global South researchers. Recognizing locally produced knowledge in research evaluations will also help global standards reflect diverse cultural realities.

## Strengths and Limitations

Though we noticed some possible progress, our findings align with earlier reviews that have highlighted persistent gaps in gender reporting practices across psychological studies. For example, Hartung and Lefler [35] found that studies often include male and female participants but fail to stratify data by gender or discuss its relevance. McHugh et al. [59] also noted that even when gender was mentioned, its analytical relevance was often unclear or omitted altogether. Cameron and Stinson [17] showed that non-binary identities were rarely acknowledged across leading psychology publications. Our contribution extends prior literature by not only evaluating whether non-binary identity was reported but also documenting how it was reported. By quantifying the terminologies that researchers used, we highlighted the language norms that shape inclusion and revealed how vague or marginalizing terms (e.g., “undisclosed,” “other”) may unintentionally obscure or stigmatize gender-diverse participants. In addition, considering insufficient gender reporting is a field-wide issue, this review focused on one, high-impact journal to enable a methodologically consistent analysis. As Cavanaugh and Abu Hussein [18] found, only 9% of psychology journals explicitly instructed authors to address gender in sections such as the abstract, introduction, or methods, and none required such information in results or discussion. In this context, a detailed, focused examination of one flagship journal provides a valuable case study of how gender reporting practices are evolving under current editorial standards. This approach allows for greater analytical depth and serves as a necessary foundation for future cross-journal comparisons. Also, we compared regional differences in gender reporting. This shed light on how cultural, institutional, and policy contexts shape research practices.

This review has limitations. First, as this review was descriptive, the findings should be interpreted as indicative rather than definitive. Confidence intervals are presented to illustrate variability rather than firm conclusions about year-to-year changes. Because the review includes publications only up to 2024 and for some comparisons the number of studies was quite low, it remains uncertain whether the observed fluctuations reflect temporary deviations or longer-term trends. Extending the analysis in future years and incorporating larger datasets would provide a clearer understanding of the evolving patterns of gender reporting in psychological research. Second, our analysis was limited to one journal. While this facilitates tracking, the findings may not be fully representative of the broader field. Additionally, since *Psychological Science* is progressive in promoting bias-free research, the insufficient gender reporting practices in Psychological Science suggests these practices may be further limited in other journals. Broader comparisons across journals such as *Annual Review of Psychology* and *Psychological Bulletin* which have different publication policies could better isolate editorial factors that influence gender reporting. Third, we did not examine whether gender-related analyses were preregistered. While preregistration can help determine whether gender was planned as an analytic variable, many articles did not report sufficient information to allow consistent evaluation of preregistration content. Future reviews could examine preregistration records directly to explore whether gender-related analyses are hypothesis-driven or added post hoc. Fourth, this review could not account for the years in which the underlying data were collected. Although the year of data collection would provide valuable context for interpreting gender reporting practices, this information was rarely reported or inconsistently described in the articles reviewed. Consequently, it was not possible to determine whether the observed decline in gender-stratified reporting in 2024 reflects recent shifts or research conducted under earlier conditions, such as pandemic-related disruptions or evolving preregistration norms. Linking publication data to data collection periods in future studies would help clarify whether 2024 represents an outlier or part of a broader trend, and enhance understanding of how methodological and policy changes shape gender reporting over time. Fifth, although the study classified research by the Global North and South, we did not examine the religious affiliations of institutions where studies were approved. Religion may influence institutional culture and researchers’ willingness to engage in gender-equitable practices. Research has found that institutions affiliated with conservative religious traditions often restrict the use of non-binary or gender-inclusive language [75, 76]. While institutional affiliation is an imperfect proxy for religious influence and some institutions retain religious names without active religious governance, future research could explore how religious and cultural contexts shape gender reporting in psychological research.

## Conclusions

In summary, this review highlights ongoing inconsistencies in how psychological researchers report gender over years and across countries. While some progress has been made, especially in recognizing non-binary identities, gender remains unevenly addressed across regions and time. These findings underscore the need for continued attention to transparent and inclusive reporting practices. Broader comparisons across journals and longitudinal monitoring may help clarify whether the patterns observed here represent temporary fluctuations or more sustained trends. Strengthening journal policies, increasing funding for gender-diverse research, and fostering interdisciplinary collaboration can promote more inclusive methodologies. And stronger international cooperation, capacity-building initiatives, and localized adaption of research guidelines help address regional disparities. Advancing gender reporting is not simply a matter of technical compliance. It is essential to producing more accurate, equitable, and socially relevant knowledge that reflects the diversity of human experience.

## Supplementary Information

Additional file 1.

## Data Availability

The datasets used and/or analysed during the current study are available from the corresponding author on reasonable request.

## Abbreviations

APA: The American Psychological Association
SAGER: The Sex and Gender Equity in Research
APS: The Association for Psychological Science
OSF: Open Science Framework
PRISMA: The Preferred Reporting Items for Systematic reviews and Meta-Analyses
EDI: Equity, diversity, and inclusion
